# Age-specific agreement between Hi-DongDong and UTAP-2 articulation assessments in Korean children aged 2–13 years

**DOI:** 10.3389/fped.2026.1944143

**Published:** 2026-10-12

**Authors:** Jinju Lee, Sejin Kwon, Jin Young Ko, Yulhyun Park, Jaewon Lee, Ju Seok Ryu, Jee Hyun Suh

**Affiliations:** 1 Department of Rehabilitation Medicine, Seoul National University Bundang Hospital, Seongnam, Republic of Korea; 2 Department of Rehabilitation, Gyeonggi Provincial Medical Center Anseong, Anseong, Republic of Korea; 3 Department of Rehabilitation, Gyeonggi Provincial Medical Center Icheon, Icheon, Republic of Korea

**Keywords:** agreement, articulation assessment, bland–Altman analysis, digital health, intraclass correlation coefficient, pediatric, speech sound disorders, telerehabilitation

## Abstract

**Introduction:**

Digital approaches to articulation assessment are increasingly proposed to expand access to speech-language evaluation, yet the comparability of speech performance elicited by digital and conventional word lists across age groups remains insufficiently understood.

**Methods:**

This study assessed age-specific agreement between Hi-DongDong word list, a novel digital articulation assessment instrument, and the Urimal Test of Articulation and Phonology 2 (UTAP-2), the standard reference in South Korea, in 505 typically developing Korean children aged 2-13 years. Each child's audio recordings were scored by three independent speech-language pathologists on both instruments. Agreement for consonant accuracy measures-Percentage of Consonants Correct (PCC), its revised form, and their totals-and for whole-word correctness was evaluated using intraclass correlation coefficients (ICC), Bland-Altman bias and limits of agreement (LoA), and linear mixed-effects models.

**Results:**

Inter-rater reliability was excellent for all metrics (ICC ≥ 0.995). At the population level, no significant instrument-by-age or instrument-by-age² interactions were observed, indicating that the age-related developmental trajectories did not differ significantly between Hi-DongDong and UTAP-2, although PWC showed a significant main effect of instrument, with lower scores for Hi-DongDong than UTAP-2 at the mean-centered age. Individual-level agreement, however, was strongly age-dependent. Low ICC values at ages 9 and 12 co-occurred with narrow LoA and ceiling-compressed scores, identifying them as artifacts of variance restriction rather than true disagreement. In contrast, ages 4 and 7 showed low ICC with wide LoA, reflecting genuine measurement divergence during periods of rapid or transitional phonological development. Among school-age children (7 years and older), bias was negligible (within ±1 percentage point) and LoA were narrow (approximately 8-24 percentage points), whereas preschool-age children showed substantially wider individual-level LoA. Hi-DongDong additionally sustained discriminative sensitivity for both consonants and vowels-including diphthongs-across a wider developmental range than the monophthong-only UTAP-2.

**Discussion:**

These findings support the use of Hi-DongDong as a complementary articulation word-list assessment across development, while underscoring that the present study evaluated clinician-scored agreement between word lists rather than automated diagnostic performance.

**Clinical Trial Registration:**

https://clinicaltrials.gov/study/NCT07273370?term=NCT07273370&rank=1, NCT07273370

## 1 Introduction

Early identification and intervention are critical, as unresolved speech sound disorders (SSDs) are associated with impaired literacy development and long-term academic difficulties (1). However, access to timely, high-quality speech-language assessment remains unevenly distributed. Families in underserved or geographically remote areas face substantial barriers, including limited availability of trained speech-language pathologists (SLPs), high out-of-pocket costs, and the logistical burden of repeated in-person clinic visits (2, 3). These disparities underscore the urgent need for scalable, accessible diagnostic alternatives that can deliver reliable assessments beyond traditional clinical settings.

Telerehabilitation and digital health technologies have emerged as promising solutions to address these systemic inequities. Recent advances in artificial intelligence (AI) and automated speech processing have accelerated the development of digital articulation assessment tools capable of delivering objective, standardized evaluations remotely. Within this context, Hi-DongDong was developed as a novel digital articulation assessment instrument by a multidisciplinary expert panel of 23 clinicians using a three-round Delphi process, incorporating 35 target words optimized for digital and AI-mediated evaluation (4).

Despite this potential, the validation of digital health assessment tools at the overall population level may obscure meaningful variation in measurement performance across clinically distinct subgroups. A previous study noted that lack of statistical heterogeneity does not necessarily exclude clinically meaningful subgroup differences, as clinically relevant heterogeneity may remain obscured within overall pooled results (5). Consistent with this perspective, Wagner et al. reported that remote physical examination using a mobile medical device demonstrated satisfactory concordance with in-person examination in children older than 2 years (6); however, performance limitations were noted in specific age groups, particularly infants, suggesting that age-related factors may critically influence the validity of digital assessments in pediatric populations.

More broadly, evidence-based analyses of the diagnostic accuracy of digital health applications remain scarce (7), and the scientific basis of many tools has not been rigorously evaluated across population subgroups. To date, no study has systematically examined whether agreement between a digital articulation assessment tool and a standardized reference instrument varies as a function of age in typically developing Korean-speaking children.

This gap is particularly consequential in pediatric populations, given the rapid and nonlinear trajectory of speech sound development across childhood. Articulation accuracy and phonological error patterns change substantially from the toddler period through school age, with key developmental transitions occurring around ages 2–3 years (word-level stage), 3–6 years (pre-school stage), and 7 years and beyond (school-age stage). Consequently, the degree to which a digital tool agrees with a standardized reference instrument may differ substantially across developmental stages. A tool that demonstrates acceptable agreement at the population level may nonetheless exhibit systematic discrepancies in specific age groups, with direct implications for clinical decision-making, diagnostic accuracy, and treatment planning.

Therefore, this study aimed to examine age-specific agreement between Hi-DongDong and the Urimal Test of Articulation and Phonology 2 (UTAP-2) word lists in typically developing Korean children aged 2–13 years. The findings are intended to provide age-stratified evidence on the comparability of speech performance elicited by the two word lists under clinician-based scoring.

## 2 Methods

### 2.1 Study design

This study enrolled typically developing children aged 2–13 years without a history of language delay or health concerns. Prior to participation, caregivers reported demographic information, including date of birth, sex, and residential region. Children's articulation was assessed using both UTAP-2 and Hi-DongDong.

### 2.2 Study procedure

Assessments were conducted in quiet, individual spaces at participants' homes or research facilities. Each target word was produced under the supervision of a caregiver or therapist, and all utterances were audio-recorded in a single session under standardized conditions across all sites.

### 2.3 Instruments

UTAP-2 is the most widely used standardized instrument for SSD assessment in South Korea, comprising 30 target words designed to evaluate 19 consonants and 7 monophthong vowels across four syllable positions (8). It provides normative data for children aged 2 to 7; however, the assessment can also be administered to children older than 8 years when evaluation of speech sound ability is clinically indicated. Despite its widespread clinical adoption, UTAP-2 relies heavily on face-to-face administration and lacks automated or digitalized scoring systems, posing challenges for accessibility, perceptual evaluation consistency, and regional equity in service delivery (9).

To address these limitations, Hi-DongDong was developed as a novel digital articulation assessment instrument through a three-round modified Delphi process. The expert panel comprised 23 multidisciplinary clinicians—12 physiatrists and 11 speech-language pathologists—selected based on relevant advanced degrees and a minimum of 5 years of clinical experience (mean professional experience: 10.69 years). The final instrument comprises 35 target words covering a broad range of Korean phonemes across word positions, with particular emphasis on phonological coverage, reduction of item ambiguity, and suitability for AI-mediated digital evaluation (4). Consensus regarding the appropriateness and content validity of the assessment items was determined using predefined criteria, including a content validity ratio ≥0.39, content validity index ≥0.78, median ≥3.5, and interquartile range ≤1.0, with items retained only when all criteria were satisfied. This consensus process reflects the Delphi-based item-development stage of the Hi-DongDong instrument and is distinct from the inter-rater reliability of the present validation study (ICC ≥0.995; Section 3.3).

The Hi-DongDong and UTAP-2 word lists are largely distinct, with only seven overlapping words. Nevertheless, both include the full set of Korean onset consonants and the major coda consonant categories. Differences between the lists are mainly related to lexical composition and to the distribution of target consonants across words and syllable positions.

### 2.4 Assessment procedure

Audio recordings were anonymized and distributed asynchronously to three independent SLPs (mean clinical experience: 12.3 ± 7.51 years, including two doctoral-level experts with first-class clinical certification and one doctoral candidate with second-class certification). Rigorous allocation concealment was enforced to prevent evaluator bias. Each evaluator independently assessed both instrument datasets once per participant, yielding six independent evaluations per child. Manual scoring was adopted for both instruments to enable direct comparison under identical scoring conditions; automated scoring algorithms for Hi-DongDong are currently under development and will be incorporated in future studies.

### 2.5 Ethical considerations

The study protocol was reviewed and approved by the Institutional Review Board (approval number: B-2510-1000-306). All participants were informed about the purpose, content, and voluntary nature of the study before participation. Written informed consent was obtained from all caregivers prior to assessment. All collected data were kept confidential and used only for research purposes in accordance with IRB-approved protocols.

### 2.6 Outcome measures

Each rater listened to the audio recordings of each child and recorded error responses for designated consonants and vowels within target words. Outcome measures included consonant accuracy metrics — Percentage of Consonants Correct (PCC), Percentage of Consonants Correct-Revised (PCC-R), Total PCC, and Total PCC-R — word-level phonological metrics — Proportion of Whole-Word Correctness (PWC) and Phonological Mean Length of Utterance (PMLU) — and a vowel accuracy metric — Percentage of Vowels Correct (PVC). PCC was calculated as the proportion of consonants produced correctly, with distortions classified as errors. PCC-R was calculated using the same method, except that distortions were classified as correct productions. Total PCC and Total PCC-R were calculated using the same criteria as PCC and PCC-R, respectively, but included all consonants within the target words rather than only designated consonants. PWC reflects the proportion of words produced without any phonological error. PMLU was calculated by combining the number of segments produced with the number of consonants produced correctly within each word, thereby reflecting both phonological complexity and production accuracy at the word level (10). PVC was calculated as the proportion of designated vowels produced correctly. Note that UTAP-2 includes only monophthongs in its word list, while Hi-DongDong includes both monophthongs and diphthongs.

### 2.7 Statistical analysis

All analyses were conducted using R (version 4.5.2). Descriptive statistics including mean, standard deviation, and range were calculated for all outcome measures by age and instrument.

Inter-rater reliability was assessed using two-way mixed-effects intraclass correlation coefficients (ICC) with absolute agreement and average-measures units for all metrics across both instruments.

First, inter-instrument agreement was examined using two-way mixed-effects ICC with absolute agreement and single-measures units, calculated by individual age and by age group (preschool: 2–6 years; school-age: 7–13 years) for PCC, PCC-R, Total PCC, Total PCC-R and PWC. ICC values were interpreted as poor (<.50), moderate (0.50–0.75), good (0.75–0.90), or excellent (≥0.90) following established guidelines (11).

Second, Bland-Altman analysis was conducted to examine systematic bias and limits of agreement (LoA) at the individual level. Bias was calculated as the mean difference between Hi-DongDong and UTAP-2 scores (Hi-DongDong minus UTAP-2), with LoA defined as bias ±1.96 standard deviation of the differences.

Third, to examine whether agreement between the instruments varied with age, linear mixed-effects models (LMMs) were fitted separately for PCC, Total PCC, and PWC. Instrument score was modeled as the outcome, with instrument type, age, sex, and residential region (metropolitan vs. non-metropolitan) entered as fixed effects and participant included as a random intercept. For each child and instrument, the mean score across the three raters was used, yielding one observation per child per instrument. To account for nonlinear developmental trajectories, both linear and quadratic age terms, together with their interactions with instrument type, were included. Linear and quadratic age specifications were compared using maximum likelihood estimation and a likelihood-ratio test, and the final model parameters were estimated using restricted maximum likelihood.

PMLU and PVC were reported descriptively but excluded from all agreement analyses (ICC, Bland-Altman, and linear mixed models). PMLU was excluded due to differences in word-list length between instruments (Hi-DongDong: 35 words; UTAP-2: 30 words), which preclude meaningful direct comparison of absolute PMLU values. PVC was excluded as UTAP-2 demonstrated complete ceiling effects (mean = 100%, SD = 0%) in school-age groups (ages 9–13 years), precluding reliable statistical computation in age-stratified analyses.

## 3 Results

### 3.1 Demographic information of participants

A total of 505 typically developing children were included (Table 1). The mean age of participants was 7.45 ± 2.52 years. Of the total sample, 50.3% were boys and 49.6% were girls, with 37.2% residing in metropolitan areas and 62.7% in non-metropolitan areas. Age distribution ranged from 2 to 13 years, with the largest subgroups at ages 7 (*n* = 93) and 6 (*n* = 69). Sample sizes were smallest at ages 2 (*n* = 7) and 13 (*n* = 10) (Table 2).

**Table 1:** Overall demographic characteristics.

Characteristics	Values
Age(yr), mean ± SD [range]	7.497 ± 2.515 [2∼13]
Sex, *n* (%)	
Boy	254 (50.3)
Girl	251 (49.7)
Region, *n* (%)	
Metropolitan (Seoul, Gyeong-Gi, Incheon)	188 (37.2)
Non-metropolitan (non-SGI)	317 (62.7)

**Table 2:** Demographic characteristics by age group.

Age (yr)	*N*	Sex		Region	
		Boy	Girl	Metropolitan	Non-metropolitan
		*N* (%)	*N* (%)	*N* (%)	*N* (%)
2	7	6 (85.7%)	1 (14.3%)	3 (42.9%)	4 (57.1%)
3	17	9 (52.9%)	8 (47.1%)	6 (35.3%)	11 (64.7%)
4	35	20 (57.1%)	15 (42.9%)	5 (14.3%)	30 (85.7%)
5	53	28 (52.8%)	25 (47.2%)	15 (28.3%)	38 (71.7%)
6	69	36 (52.2%)	33 (47.8%)	27 (39.1%)	42 (60.9%)
7	93	44 (47.3%)	49 (52.7%)	43 (46.2%)	50 (53.8%)
8	57	24 (42.1%)	33 (57.9%)	21 (36.8%)	36 (63.2%)
9	68	36 (52.9%)	32 (47.1%)	26 (38.2%)	42 (61.8%)
10	36	19 (52.8%)	17 (47.2%)	11 (30.6%)	25 (69.4%)
11	27	11 (40.7%)	16 (59.3%)	13 (48.1%)	14 (51.9%)
12	33	15 (45.5%)	18 (54.5%)	11 (33.3%)	22 (66.7%)
13	10	6 (60%)	4 (40%)	7 (70%)	3 (30%)
Total	505	254	251	188	317

### 3.2 Descriptive statistics of PCC, PCC_R, PCC Total, PCC_R Total, PWC, PMLU, PVC

Descriptive statistics for all outcome measures are presented in Table 3. Ceiling effects were observed for PCC, PCC-R, Total PCC, Total PCC-R, and PVC among children aged 9–13 years. With the exception of PMLU and PVC measured using UTAP-2, mean scores at age 2 (*n* = 7) were higher than those at age 3 (*n* = 17) across all instruments and outcome measures; however, this pattern should be interpreted with caution given the small sample size at age 2. From age 3 years onward, mean scores for PCC, PCC-R, and PWC showed a generally increasing trend through age 9 years (*n* ≥ 35), while Total PCC and Total PCC-R increased through age 10 years (*n* ≥ 35). PMLU showed an increasing trend from age 3 to 6 years, and PVC from age 3 to 4 years. Notably, all measures demonstrated a marked increase in mean scores between ages 3 and 4 (Figure 1).

**Table 3:** Descriptive statistics.

Age (yr)	Instrument	*n*	Descriptive Statistics: Mean ± SD (Range)						
			PCC	PCC_R	PCC Total	PCC Total_R	PWC	PMLU	PVC
2^a^	HiDD	7	79.5 ± 15.4 (54.2–95.8)	79.5 ± 15.4 (54.2–95.8)	79.7 ± 14.9 (55.4–94)	79.7 ± 14.9 (55.4–94)	54.3 ± 20.2 (17.1–80)	6.6 ± 0.5 (5.8–6.9)	87.6 ± 7.7 (73.9–95.7)
	U2	7	78 ± 14.4 (47.9–87.5)	78 ± 14.4 (47.9–87.5)	77.4 ± 20.3 (33.3–89.4)	77.4 ± 20.3 (33.3–89.4)	56.2 ± 20.3 (16.7–76.7)	7.7 ± 1 (5.6–8.2)	90.5 ± 12.6 (71.4–100)
3^a^	HiDD	17	74.7 ± 13.8 (50–97.9)	74.7 ± 13.8 (50–97.9)	74 ± 14 (50.6–98.8)	74 ± 14 (50.6–98.8)	45.1 ± 20.8 (17.1–85.7)	6.3 ± 0.5 (5.5–7.1)	80.3 ± 10.2 (60.9–95.7)
	U2	17	72.8 ± 14.6 (50–96.5)	72.8 ± 14.6 (50–96.5)	71.5 ± 15.7 (45–95.7)	71.5 ± 15.7 (45–95.7)	43.2 ± 22.7 (6.7–90)	7.3 ± 0.8 (5.9–8.4)	91 ± 14.2 (47.6–100)
4	HiDD	35	89.4 ± 13.9 (27.1–100)	89.4 ± 13.9 (27.1–100)	88.4 ± 14.6 (25.3–100)	88.4 ± 14.6 (25.3–100)	71.9 ± 22 (5.7–100)	6.8 ± 0.5 (4.7–7.2)	89.9 ± 9.6 (60.9–100)
	U2	35	90.4 ± 10.6 (60.4–100)	90.5 ± 10.6 (60.4–100)	89.4 ± 11.9 (51.4–100)	89.5 ± 11.9 (51.4–100)	74 ± 23.9 (20–100)	8.2 ± 0.5 (6.2–8.6)	99.6 ± 2.4 (85.7–100)
5	HiDD	53	93.9 ± 8.2 (66.7–100)	93.9 ± 8.2 (66.7–100)	93.6 ± 8 (63.9–100)	93.6 ± 8 (63.9–100)	81.3 ± 15.9 (42.9–100)	7 ± 0.3 (6–7.2)	93 ± 6.9 (78.3–100)
	U2	53	94.6 ± 8.1 (68.8–100)	94.6 ± 8.1 (68.8–100)	94.4 ± 8.1 (69.1–100)	94.4 ± 8.1 (69.1–100)	85.6 ± 17.4 (33.3–100)	8.4 ± 0.4 (6.9–8.6)	98.9 ± 3.8 (85.7–100)
6	HiDD	69	96.8 ± 4.9 (75–100)	96.8 ± 4.9 (75–100)	96.5 ± 5.1 (75.9–100)	96.5 ± 5.1 (75.9–100)	87.3 ± 12.9 (48.6–100)	7.1 ± 0.1 (6.6–7.2)	94.8 ± 6.1 (78.3–100)
	U2	69	95.8 ± 6 (70.8–100)	95.8 ± 6 (70.8–100)	96.2 ± 5.3 (75.5–100)	96.2 ± 5.3 (75.5–100)	89.2 ± 13.7 (43.3–100)	8.5 ± 0.2 (7.4–8.6)	98.8 ± 4.1 (85.7–100)
7	HiDD	93	97.9 ± 2.9 (88.2–100)	97.9 ± 2.9 (88.2–100)	97.7 ± 2.4 (89.6–100)	97.7 ± 2.4 (89.6–100)	91.8 ± 7.6 (62.9–100)	7.1 ± 0.1 (6.8–7.2)	97.1 ± 4.6 (73.9–100)
	U2	93	98 ± 2.8 (87.5–100)	98 ± 2.8 (87.5–100)	98.2 ± 2.2 (89.4–100)	98.2 ± 2.2 (89.4–100)	94.3 ± 6.8 (66.7–100)	8.5 ± 0.1 (8.2–8.6)	99.8 ± 1.5 (85.7–100)
8	HiDD	57	98.4 ± 2.6 (89.6–100)	98.4 ± 2.6 (89.6–100)	98.3 ± 2.5 (90.4–100)	98.3 ± 2.5 (90.4–100)	94.4 ± 6.4 (74.3–100)	7.1 ± 0.1 (6.9–7.2)	98.4 ± 3.1 (87–100)
	U2	57	98.2 ± 3.8 (77.1–100)	98.2 ± 3.8 (77.1–100)	98.4 ± 3.2 (81.9–100)	98.4 ± 3.2 (81.9–100)	95 ± 8.8 (53.3–100)	8.5 ± 0.1 (8–8.6)	99.7 ± 1.9 (85.7–100)
9	HiDD	68	99.1 ± 1.3 (95.8–100)	99.1 ± 1.3 (95.8–100)	99.1 ± 1.1 (96.4–100)	99.1 ± 1.1 (96.4–100)	96.4 ± 3.4 (87.6–100)	7.2 ± 0 (7.1–7.2)	98.6 ± 2.2 (91.3–100)
	U2	68	98.6 ± 2.4 (88.2–100)	98.6 ± 2.4 (88.2–100)	98.9 ± 1.7 (92.9–100)	98.9 ± 1.7 (92.9–100)	96.5 ± 5.1 (77.8–100)	8.6 ± 0.1 (8.3–8.6)	100 ± 0 (100–100)
10	HiDD	36	99.1 ± 2.2 (91–100)	99.1 ± 2.2 (91–100)	99.3 ± 1.7 (93.6–100)	99.3 ± 1.7 (93.6–100)	96.1 ± 4.9 (78.1–100)	7.2 ± 0.1 (7–7.2)	98.3 ± 3.3 (87–100)
	U2	36	99.2 ± 1.6 (93.8–100)	99.2 ± 1.6 (93.8–100)	99.2 ± 1.7 (92.6–100)	99.2 ± 1.7 (92.6–100)	97.8 ± 4.2 (83.3–100)	8.6 ± 0.1 (8.3–8.6)	100 ± 0 (100–100)
11^a^	HiDD	27	98.6 ± 2.5 (88.9–100)	98.6 ± 2.5 (88.9–100)	98.6 ± 2.9 (87.5–100)	98.6 ± 2.9 (87.5–100)	95.8 ± 6.1 (74.3–100)	7.1 ± 0.1 (6.9–7.2)	98.5 ± 2.9 (91.3–100)
	U2	27	98.3 ± 2.9 (87.5–100)	98.3 ± 2.9 (87.5–100)	98.8 ± 2.1 (91.9–100)	98.8 ± 2.1 (91.9–100)	96.2 ± 6.8 (74.4–100)	8.6 ± 0.1 (8.3–8.6)	100 ± 0 (100–100)
12^a^	HiDD	33	99.6 ± 0.9 (97.9–100)	99.6 ± 0.9 (97.9–100)	99.5 ± 0.7 (97.6–100)	99.5 ± 0.7 (97.6–100)	97.7 ± 2.6 (88.6–100)	7.2 ± 0 (7.1–7.2)	99 ± 2.2 (91.3–100)
	U2	33	99.3 ± 1.2 (95.8–100)	99.3 ± 1.2 (95.8–100)	99.3 ± 1.3 (94.7–100)	99.3 ± 1.3 (94.7–100)	97.8 ± 3.4 (90–100)	8.6 ± 0.1 (8.4–8.6)	100 ± 0 (100–100)
13	HiDD	10	98.8 ± 2 (93.8–100)	98.8 ± 2 (93.8–100)	99 ± 1.5 (95.2–100)	99 ± 1.5 (95.2–100)	96.6 ± 3.8 (88.6–100)	7.2 ± 0 (7.1–7.2)	98.7 ± 2.1 (95.7–100)
	U2	10	96.5 ± 6.5 (79.9–100)	96.6 ± 6.3 (80.6–100)	97.6 ± 4.5 (85.8–100)	97.7 ± 4.3 (86.5–100)	93.7 ± 10.8 (66.7–100)	8.5 ± 0.2 (7.9–8.6)	100 ± 0 (100–100)

a Age groups with *n* < 30 are highlighted in red; ICC estimates for these groups may be unstable due to small sample sizes and should be interpreted with caution.

HiDD, Hi-DongDong; U2, UTAP-2.

**Figure 1:**
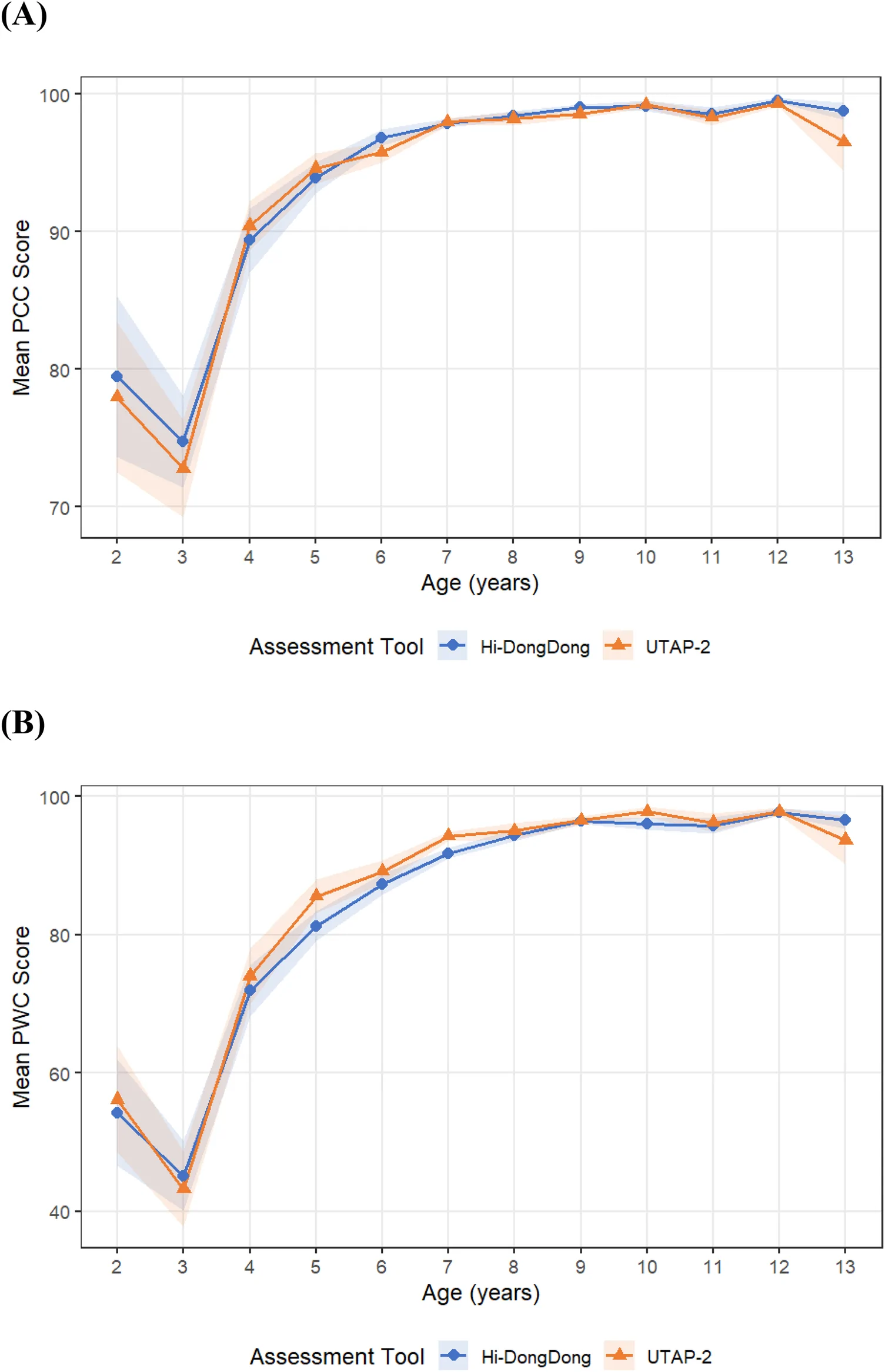
Age-related trajectories of articulation and phonological accuracy measured by Hi-DongDong and UTAP-2. **(A)** Mean percentage of consonants correct (PCC) and **(B)** mean percentage of whole-word correctness (PWC) by chronological age in typically developing Korean children aged 2–13 years.

Regarding instrument-specific developmental trajectories, mean PCC scores in Hi-DongDong showed no further increase after age 9 years (99.1 ± 1.3%), whereas UTAP-2 scores continued to increase slightly through age 10 years (99.2 ± 1.6%). In contrast, for PVC, UTAP-2 mean scores showed no further meaningful increase after age 4 years (99.6 ± 2.4%), while Hi-DongDong scores continued to increase gradually through age 9 years (98.6 ± 2.2%).

### 3.3 Inter-rater reliability

Inter-rater reliability was excellent for all metrics across both instruments (ICC ≥0.995) (Table 4). Review of individual rater data revealed that two of three raters recorded no distortion errors across both instruments, while one rater identified a small number of distortions (Hi-DongDong: PCC-R *n* = 2, Total PCC-R *n* = 2; UTAP-2: PCC-R *n* = 6, Total PCC-R *n* = 8). Despite these minor differences in distortion classification, inter-rater reliability for PCC-R and Total PCC-R remained excellent (ICC ≥0.999).

**Table 4:** Inter-rater reliability.

Test type	Metric	ICC [95% CI]
HiDD	PCC	1 [0.999, 1]
	PCC_R	1 [0.999, 1]
	Total_PCC	1 [1, 1]
	Total_PCC_R	1 [1, 1]
	PWC	1 [1, 1]
	PMLU	1 [1, 1]
	PVC	0.999 [0.999, 0.999]
UTAP2	PCC	0.999 [0.999, 0.999]
	PCC_R	0.999 [0.999, 1]
	Total_PCC	0.999 [0.999, 1]
	Total_PCC_R	1 [1, 1]
	PWC	0.999 [0.999, 1]
	PMLU	1 [1, 1]
	PVC	0.995 [0.994, 0.995]

### 3.4 Age-specific inter-instrument agreement between Hi-DongDong and UTAP-2

Age-specific inter-instrument agreement results, including ICC values and Bland–Altman analyses, are presented in Table 5. ICC values and Bland–Altman LoA varied substantially across in age group. Among preschool-age children, ages 2 and 3 demonstrated good agreement for all consonant accuracy measures (PCC and Total PCC: ICC = 0.78–0.89), with relatively wide LoA ranges (e.g., age 2 PCC: −21.85 to 18.82; age 3 PCC: −17.11 to 13.20). PWC at age 2 showed excellent agreement (ICC = 0.987). Age 4, however, showed the lowest ICC values across all age groups for consonant accuracy measures (PCC: ICC = 0.42; Total PCC: ICC = 0.46), accompanied by the widest LoA ranges observed in the entire dataset (PCC: −25.14 to 27.29; PWC: −38.71 to 42.88). Ages 5 and 6 showed moderate agreement for PCC and PWC (ICC = 0.72–0.76), with progressively narrower LoA compared to younger ages (age 6 PCC: −8.88 to 6.74).

**Table 5:** Age-specific inter-instrument agreement (ICC and bland-altman) between Hi-DongDong and UTAP-2.

Age (yr)	Metric	*N*	ICC [95% CI]	Agreement	Bias	95% LoA [lower, upper]
2	PCC	7	0.781 [0.142, 0.959]	Good	−1.51	[−21.85, 18.82]
	Total PCC		0.857 [0.401, 0.974]	Good	−2.32	[−21.73, 17.09]
	PWC		0.987 [0.920, 0.998]	Excellent	1.90	[−3.63, 7.43]
3	PCC	17	0.851 [0.644, 0.943]	Good	−1.95	[−17.11, 13.20]
	Total PCC		0.885 [0.713, 0.957]	Good	−2.54	[−16.10, 11.01]
	PWC		0.886 [0.718, 0.957]	Good	−1.95	[−22.55, 18.65]
4	PCC	35	0.420 [0.102, 0.659]	Poor	1.07	[−25.14, 27.29]
	Total PCC		0.463 [0.154, 0.688]	Poor	1.04	[−26.17, 28.25]
	PWC		0.595 [0.330, 0.773]	Moderate	2.09	[−38.71, 42.88]
5	PCC	53	0.723 [0.565, 0.830]	Moderate	0.69	[−11.17, 12.55]
	Total PCC		0.729 [0.574, 0.834]	Moderate	0.76	[−10.82, 12.34]
	PWC		0.741 [0.571, 0.846]	Moderate	4.30	[−18.22, 26.81]
6	PCC	69	0.726 [0.588, 0.822]	Moderate	−1.07	[−8.88, 6.74]
	Total PCC		0.805 [0.703, 0.874]	Good	−0.33	[−6.71, 6.06]
	PWC		0.758 [0.637, 0.843]	Moderate	1.83	[−16.15, 19.80]
7	PCC	93	0.260 [0.059, 0.440]	Poor	0.09	[−6.68, 6.86]
	Total PCC		0.346 [0.157, 0.512]	Poor	0.47	[−4.71, 5.64]
	PWC		0.373 [0.185, 0.535]	Poor	2.48	[−13.06, 18.01]
8	PCC	57	0.602 [0.407, 0.745]	Moderate	−0.18	[−5.93, 5.57]
	Total PCC		0.754 [0.615, 0.847]	Good	0.05	[−3.89, 4.00]
	PWC		0.667 [0.493, 0.789]	Moderate	0.63	[−11.70, 12.96]
9	PCC	68	0.388 [0.172, 0.570]	Poor	−0.49	[−4.66, 3.67]
	Total PCC		0.275 [0.041, 0.481]	Poor	−0.19	[−3.53, 3.15]
	PWC		0.265 [0.027, 0.473]	Poor	0.12	[−10.27, 10.52]
10	PCC	36	0.753 [0.567, 0.866]	Good	0.11	[−2.56, 2.78]
	Total PCC		0.703 [0.488, 0.837]	Moderate	−0.05	[−2.62, 2.53]
	PWC		0.593 [0.320, 0.772]	Moderate	1.68	[−6.06, 9.41]
11	PCC	27	0.801 [0.611, 0.904]	Good	−0.28	[−3.66, 3.10]
	Total PCC		0.841 [0.683, 0.924]	Good	0.22	[−2.63, 3.07]
	PWC		0.799 [0.607, 0.903]	Good	0.42	[−7.71, 8.55]
12	PCC	33	0.314 [−0.021, 0.588]	Poor	−0.25	[−2.72, 2.21]
	Total PCC		0.416 [0.093, 0.661]	Poor	−0.20	[−2.43, 2.02]
	PWC		0.522 [0.217, 0.732]	Moderate	0.08	[−5.72, 5.89]
13	PCC	10	0.494 [−0.077, 0.839]	Poor	−2.22	[−11.36, 6.92]
	Total PCC		0.532 [−0.036, 0.854]	Moderate	−1.42	[−7.57, 4.73]
	PWC		0.435 [−0.192, 0.819]	Poor	−2.90	[−19.72, 13.92]

PCC-R and Total PCC-R yielded ICC values identical to PCC and Total PCC, respectively (differing only in the direction of bias), and are therefore omitted; full values are provided in Supplementary Table S3.

Agreement was interpreted following Koo & Li (11): poor (<0.50), moderate (0.50–0.75), good (0.75–0.90), excellent (≥0.90). Age groups with *n* < 30 (ages 2, 3, 11, 13) yield less stable ICC estimates and should be interpreted with caution.

ICC, intraclass correlation coefficient; CI, confidence interval; LoA, limits of agreement; PCC, percentage of consonants correct; Total PCC, percentage of consonants correct across all consonants in the target words; PWC, proportion of whole-word correctness. Bias = Hi-DongDong minus UTAP-2.

Among school-age children, LoA ranges were consistently narrower than those observed in preschool groups across all measures. However, ICC values were variable. Age 11 demonstrated good agreement (PCC: ICC = 0.801), while ages 8 and 10 showed moderate agreement (PCC: ICC = 0.602 and 0.753, respectively). In contrast, ages 7, 9, and 12 showed low ICC for consonant accuracy measures (age 7 PCC: ICC = 0.260; age 9 PCC: ICC = 0.388; age 12 PCC: ICC = 0.314), despite narrow LoA ranges (e.g., age 9 PCC: −4.66 to 3.67; age 12 PCC: −2.72 to 2.21). Age 13 showed low to moderate agreement across measures (PCC: ICC = 0.494; Total PCC: ICC = 0.532–0.550) with wide 95% confidence intervals, though interpretation is limited by the small sample size (*n* = 10).

Representative Bland–Altman plots for PCC at ages 4, 7, 9, and 12 are shown in Figure 2, illustrating contrasting age-specific agreement patterns. Corresponding plots for PWC and Total PCC are provided in Supplementary Figure S1. Notably, PCC and PCC-R demonstrated identical ICC values across all age groups, as did Total PCC and Total PCC-R, with bias direction being the only difference between paired metrics.

**Figure 2:**
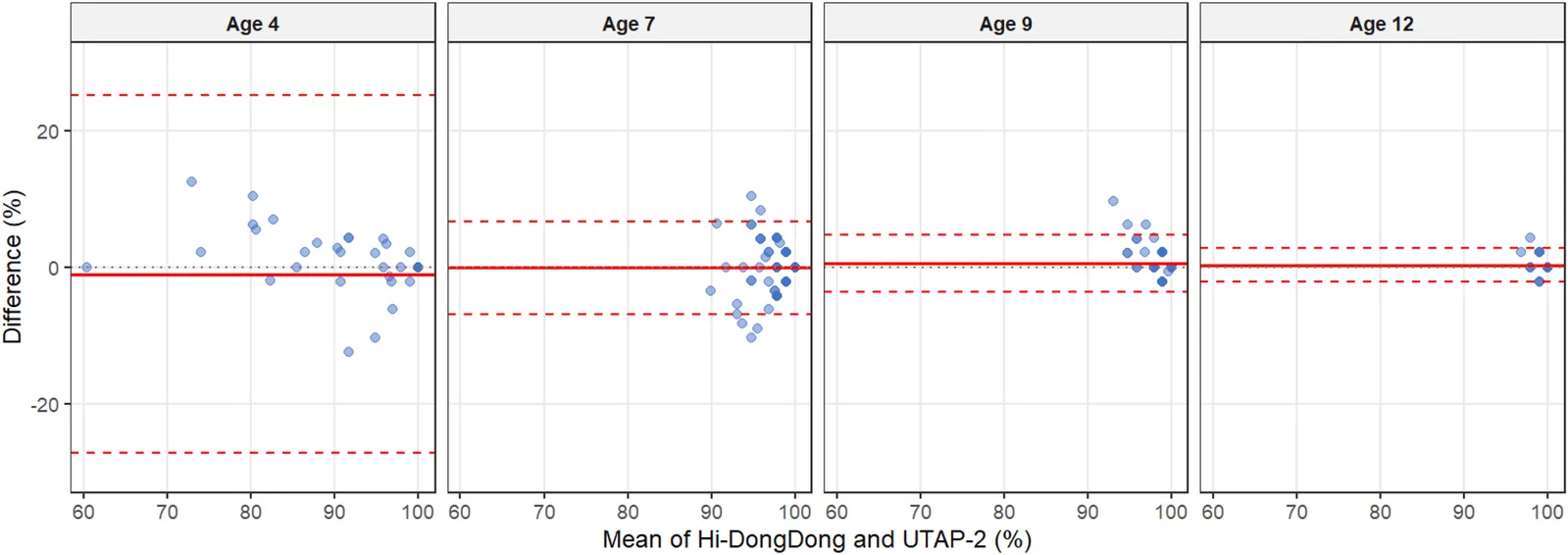
Bland-altman plots for PCC at ages 4, 7, 9, and 12 years. Difference = Hi-DongDong—UTAP-2; Solid red line: mean difference (bias); dashed red lines: 95% limits of agreement (bias ± 1.96 SD). These ages were selected to illustrate contrasting age-specific agreement patterns identified in the stratified analyses. The solid red line indicates the mean difference (Hi-DongDong—UTAP-2), and the dashed red lines indicate the 95% limits of agreement. Ages 4 and 7 showed broader dispersion of individual differences, whereas ages 9 and 12 showed restricted score variability near the upper end of the scale, consistent with ceiling effects.

### 3.5 Inter-instrument agreement across age groups

Age-specific inter-instrument agreement results are presented in Table 6. In the preschool-age group (*n* = 181), inter-instrument agreement for consonant accuracy measures ranged from moderate to good, with ICC values ranging from 0.773 (PCC) to 0.827 (PWC). However, Bland–Altman analyses revealed wide 95% LoA (ranges approximately 30–50 percentage points), indicating substantial individual-level variability between the two assessment tools. In contrast, bias values were small across all measures (within ±2.25 percentage points), suggesting that systematic differences between instruments were minimal at the group level.

**Table 6:** Group-level inter-instrument agreement (ICC and bland-altman) between Hi-DongDong and UTAP-2, by age group.

Age group	Metric	*N*	ICC [95% CI]	Agreement	Bias	95% LoA [lower, upper]
Preschool (2–6 y)	PCC	181	0.773 [0.707, 0.826]	Good	0.24	[−15.00, 15.48]
	Total PCC		0.796 [0.736, 0.844]	Good	0.03	[−15.06, 15.12]
	PWC		0.827 [0.773, 0.868]	Good	−2.25	[−27.41, 22.91]
School-age (7–13 y)	PCC	324	0.484 [0.397, 0.563]	Poor	0.22	[−5.05, 5.48]
	Total PCC		0.574 [0.496, 0.643]	Moderate	−0.05	[−4.07, 3.96]
	PWC		0.524 [0.440, 0.599]	Moderate	−0.99	[−13.08, 11.10]

Agreement was interpreted following Koo & Li (11): poor (<0.50), moderate (0.50–0.75), good (0.75–0.90), excellent (≥0.90).

ICC, intraclass correlation coefficient; CI, confidence interval; LoA, limits of agreement; PCC, percentage of consonants correct; Total PCC, percentage of consonants correct across all consonants in the target words; PWC, proportion of whole-word correctness. Bias = Hi-DongDong minus UTAP-2.

In the school-age group (*n* = 324), ICC values were lower than those observed in the preschool-age group, ranging from 0.484 (PCC) to 0.574 (Total PCC), reflecting poor-to-moderate agreement. This pattern likely reflects a ceiling effect, as reduced score variability in older children attenuates ICC estimates regardless of true instrument agreement. Notably, LoA were substantially narrower in the school-age group (approximately 8–24 percentage points) relative to the preschool-age group, suggesting greater individual-level score exchangeability between instruments. Bias remained negligible across all measures (within ±1 percentage point).

Across age groups, ICC and LoA showed divergent patterns: higher ICC but wider LoA in preschool-age children, and lower ICC but narrower LoA in school-age children. This dissociation underscores the importance of interpreting both metrics jointly, as ICC reflects relative consistency while LoA captures absolute interchangeability at the individual level.

### 3.6 Fixed and random effects from the final linear mixed-effects models predicting PCC, total PCC, and PWC

To account for the nonlinear developmental trajectories of the articulation outcomes, linear and quadratic age models were first compared using maximum likelihood estimation. For PCC, the quadratic age model provided a significantly better fit than the linear model (likelihood-ratio *χ*^2^(2) = 134.07, *p* < .001; *Δ*AIC = 130.1). The final quadratic linear mixed-effects model, which included sex and region as covariates, was therefore refitted using restricted maximum likelihood, and the results are presented in Table 7.

**Table 7:** Fixed and random effects of the final quadratic linear mixed-effects models predicting PCC, total PCC and PWC, adjusted for sex and residential region.

Predictor	PCC		Total PCC		PWC	
	b (95% CI)	*p*	b (95% CI)	*p*	b (95% CI)	*p*
Fixed effects						
Intercept	97.700 (96.653, 98.748)	<.001***	98.040 (96.960, 99.120)	<.001***	94.151 (92.116, 96.185)	<.001***
Age (centered)	1.656 (1.437, 1.874)	<.001***	1.774 (1.550, 1.997)	<.001***	3.783 (3.362, 4.204)	<.001***
Age^2^	−0.422 (−0.493, −0.350)	<.001***	−0.449 (−0.523, −0.376)	<.001***	−0.859 (−0.997, −0.721)	<.001***
Instrument: *Hi-DongDong* (ref: UTAP-2)	0.075 (−0.508, 0.657)	.802	−0.307 (−0.862, 0.248)	.278	−1.892 (−2.928, −0.857)	<.001***
Sex: *Boy* (ref: Girl)	−0.781 (−1.770, 0.208)	.122	−0.881 (−1.907, 0.145)	.093	−2.296 (−4.230, −0.361)	.020*
Region: *Non- metropolitan* (ref: Metropolitan)	2.070 (1.047, 3.094)	<.001***	1.973 (0.912, 3.035)	<.001***	3.815 (1.814, 5.816)	<.001***
Interaction						
Hi-DongDong* × age (centered)*	0.034 (−0.146, 0.214)	.710	−0.006 (−0.177, 0.165)	.943	0.276 (−0.044, 0.596)	.092
Hi-DongDong × *age*^2^	0.024 (−0.036, 0.083)	.434	0.045 (−0.011, 0.101)	.119	0.072 (−0.034, 0.177)	.183
Random effects						
*τ*^2^ (between-child variance)	25.330		28.36		101.19	
*σ*^2^ (residual variance)	13.140		11.91		41.52	
ICC	0.658		0.704		0.709	

Estimates were obtained using restricted maximum likelihood, with degrees of freedom approximated using Satterthwaite's method. Age was mean-centered (mean = __ years). *b* = unstandardized regression coefficient; SE = standard error; CI = confidence interval; Age² = quadratic term of mean-centered age; ref = reference category; τ^2^ = between-child variance; σ^2^ = residual (within-child) variance; ICC = intraclass correlation coefficient; PCC = percentage of consonants correct; UTAP-2 = Urimal Test of Articulation and Phonology-2.

* *p* < .05.

*** *p* < .001.

Age was a significant predictor of PCC (*b* = 1.656, *p* < .001), and the significant negative quadratic term based on centered age (b = −0.422, *p* < .001) indicated that PCC increased with age, but that the rate of increase progressively decelerated across the age range. Because age was mean-centered, the linear age coefficient represents the estimated change in PCC per one-year increase in age around the mean age, whereas the quadratic term captures the nonlinear curvature of the age-related trajectory. Regarding the main effect of test instrument, Hi-DongDong did not differ significantly from UTAP-2 (*b* = 0.075, *p* = .802). Among the covariates, sex was not a significant predictor (*b* = −0.781, *p* = .122), whereas region was significant (*b* = 2.070, *p* < .001), with children from non-metropolitan areas scoring approximately 2.07 percentage points higher than those from metropolitan areas.

Neither the Test × Age interaction for Hi-DongDong (*b* = 0.034, *p* = .710) nor the Test × Age^2^ interaction for Hi-DongDong (*b* = 0.024, *p* = .434) was statistically significant. These findings indicate that although PCC followed a nonlinear developmental trajectory, there was no evidence that the age-related trajectory differed significantly between Hi-DongDong and UTAP-2.

Between-child variance remained larger than residual variance (*τ*^2^ = 25.330 vs. *σ*^2^ = 13.140), indicating considerable between-child variability in PCC. The ICC of.658 indicated that approximately 65.8% of the total variance in PCC was attributable to stable between-child differences, supporting the inclusion of a participant-level random intercept.

A sensitivity analysis excluding seven observations with absolute scaled residuals greater than 3 yielded a similar overall pattern of results. Neither the Test × Age interaction (*p* = .392) nor the Test × Age^2^ interaction (*p* = .091) reached statistical significance.

For PWC, the quadratic model also provided a significantly better fit than the linear model (likelihood-ratio *χ*^2^(2) = 141.76, *p* < .001). Hi-DongDong showed a significantly lower PWC score than UTAP-2 at the mean-centered age (*b* = −1.892, *p* < .001). However, neither the Test × Age interaction (*b* = 0.276, *p* = .092) nor the Test × Age^2^ interaction (*b* = 0.072, *p* = .183) was statistically significant. Thus, although a difference in absolute PWC level was observed between the instruments, the age-related PWC trajectory did not differ significantly.

Across PCC, Total PCC, and PWC, no significant Test × Age or Test × Age^2^ interactions were observed, indicating that the age-related developmental trajectories did not differ significantly between Hi-DongDong and UTAP-2.

## 4 Discussion

This study examined age-specific inter-instrument agreement between Hi-DongDong, a novel digital articulation assessment tool, and UTAP-2, the standard reference instrument for speech sound disorder assessment in South Korea, in 505 typically developing Korean-speaking children aged 2–13 years. Inter-rater reliability was excellent across all metrics and both instruments (ICC ≥0.995), confirming that scoring variability did not confound the inter-instrument comparisons. At the population level, the quadratic linear mixed-effects models showed no significant instrument-by-age interactions for PCC, Total PCC, or PWC, indicating that the age-related developmental trajectories captured by Hi-DongDong and UTAP-2 did not differ significantly across the age range. For PCC and Total PCC, there was also no significant main effects of instrument, whereas PWC showed a significant instrument effect, with lower scores for Hi-DongDong at the mean-centered age. Thus, the two instruments showed broadly similar age-related developmental patterns despite some outcome-specific differences in absolute score levels. Against this backdrop, agreement at the individual level varied substantially by age, producing two patterns that structure the remainder of our discussion: a dissociation between ICC and Bland-Altman limits of agreement (LoA) across developmental stages, and a marked difference in absolute score agreement between preschool-age and school-age children.

An additional finding was the regional difference in PCC, Total PCC, and PWC scores, with children from non-metropolitan regions showing higher scores than those from metropolitan regions after adjustment for age, sex, and instrument type. Regional variation in Korean children's speech-sound production has also been reported previously. Shin et al. (12), using the UTAP-2 in 619 Korean children aged 2–7 years, found regional differences in PWC and consonant-accuracy measures at 2 years 6–11 months and 3 years 0–5 months, but no regional differences in any speech-production measure after 3 years 6 months (12). Because regional variation was not a primary focus of the present study, our finding should be interpreted cautiously. The observed difference may reflect unmeasured variation in linguistic environments, regional speech characteristics, recruitment characteristics, or sociodemographic factors rather than a direct effect of residential region. Given that 62.7% of participants resided in non-metropolitan regions, the regional composition of the sample should also be considered when interpreting the generalizability of the findings. Notably, the direction and persistence of the regional association in the present study differ from those reported by Shin et al. (12), suggesting that the observed regional effect may depend on sample composition, age distribution, or other unmeasured contextual factors.

Sex-related differences were outcome-specific in the present study. Sex was not significantly associated with PCC or Total PCC, whereas boys showed significantly lower PWC scores than girls. The absence of a significant sex effect for the consonant-based measures is unlikely to be explained solely by an imbalanced sex distribution, as the overall sample included nearly equal numbers of boys and girls (254 vs. 251), although the relatively small sample sizes in the youngest age groups may have limited age-specific comparisons. Previous studies also suggest that sex differences in speech-sound development are not consistently observed across ages or languages. In British English-speaking children, sex differences were absent in younger age groups but greater phonological accuracy was observed in girls in the oldest age group (13), whereas several studies of typically developing Korean children have reported significant age-related improvements in consonant and whole-word accuracy without significant overall sex differences (7, 14).

Accordingly, age- and context-related variation may have been more prominent than an overall sex effect for PCC and Total PCC in the present sample. The significant sex effect for PWC, however, suggests that sex-related differences may be more detectable at the whole-word level, where an error in any segment affects whether the entire word is scored as correct. This outcome-specific finding warrants further investigation rather than being interpreted as evidence of a general sex difference in articulation ability.

The central methodological message of this study is that ICC and LoA capture different, non-redundant facets of agreement, and that relying on either alone can be misleading. ICC reflects relative consistency—the ability to rank children similarly—and is defined as the ratio of between-subject variance to total variance. LoA, by contrast, reflects absolute interchangeability at the individual level, independent of how much true variance exists in the sample. Because ICC is mathematically dependent on between-subject variance, it is systematically attenuated whenever score variability is restricted, even when the two instruments agree closely in absolute terms. This dependence is not a peripheral statistical nuance in our data; it is the key to interpreting the age-specific results, and it explains why several age groups exhibited the counterintuitive combination of low ICC with narrow LoA. We therefore interpret ICC and LoA jointly throughout, treating a low ICC as diagnostic of poor agreement only when it is not accompanied by narrow LoA and restricted score variance.

Applying this joint interpretation resolves the apparent paradox in the age-specific findings by separating two mechanistically distinct sources of low ICC. In the first group—ages 9 and 12—low ICC coincided with narrow LoA (e.g., age 9 PCC LoA −4.66 to 3.67; age 12 PCC LoA −2.72 to 2.21) and with scores compressed into the 95%–100% range. Here, the low ICC likely reflects ceiling-induced variance restriction rather than poor inter-instrument agreement, as the narrow LoA indicate only small absolute differences between the two tools. The second group—ages 4 and 7—is qualitatively different and more informative. Age 4 showed the lowest ICC and the widest LoA of any age group (PCC ICC = 0.42; LoA −25.14 to 27.29), and age 7, despite an ample sample (*n* = 93) and no ceiling effect, showed a low and unstable ICC (PCC ICC = 0.260; lower CI bound 0.059–0.185). Because neither small sample size nor ceiling restriction can account for these values, they most plausibly reflect genuine measurement discrepancy between the instruments. The lower agreement observed at ages 4 and 7 may reflect differences in how the two word lists sample phonological performance at these ages. At age 4, consonant production is still undergoing substantial development and may show greater inter-individual variability (7). Age 7 also represents a transitional period at the boundary between preschool and school-age assessment contexts, during which differences in word-list composition may differentially affect measurement sensitivity among children approaching more mature speech patterns. In addition, articulation accuracy can vary according to the phonetic and linguistic context of elicitation words. Previous Korean studies have shown that consonant accuracy and acquisition may differ according to phonological context, word length, and the syllable position of target consonants (7, 15). Although Hi-DongDong and UTAP-2 cover broadly similar consonant inventories, the two word lists differ in lexical composition, word length, syllable structure, and the frequency and positional distribution of target consonants. These differences may therefore contribute to the lower agreement observed at ages 4 and 7, particularly when consonant production is still developing or when residual errors are restricted to specific phonological contexts. Because the contribution of each word-list characteristic was not directly tested, these factors should be regarded as plausible explanations rather than confirmed causes.

The symmetrical bias between PCC and PCC-R (and between Total PCC and Total PCC-R) further localizes the disagreement to the differential scoring of distorted consonants, rather than to gross transcription differences.

Hi-DongDong and UTAP-2 use largely distinct word lists, with only seven overlapping words, although their overall consonant inventories are broadly comparable. Because PWC reflects the proportion of words produced entirely correctly, the largely non-overlapping lexical items may contribute to the significant difference in absolute PWC scores between Hi-DongDong and UTAP-2. Even when the same consonant phonemes are represented, differences in word length, syllable structure, phonological complexity, and the frequency and positional distribution of target consonants may affect whether an entire word is scored as correct. Importantly, this difference was limited to the absolute PWC level; neither the Test × Age nor the Test × Age^2^ interaction was significant, indicating that the age-related PWC trajectory did not differ significantly between the two instruments. Because PCC and Total PCC quantify consonant accuracy at the segmental level, their similar results across instruments may be related to the broadly comparable consonant inventories represented in the two word lists. However, this interpretation should be considered cautiously because the present study did not directly test whether consonant coverage accounted for the absence of an instrument effect.

A distinct and clinically favorable pattern emerged for vowel accuracy that highlights a genuine difference in what the two instruments measure. UTAP-2 mean PVC reached ceiling as early as age 4 years (99.6 ± 2.4%) and increased no further thereafter, whereas Hi-DongDong PVC continued to increase gradually through age 9 years (98.6 ± 2.2%). This divergence is unlikely to reflect a true difference in the timing of vowel development, since diphthong acquisition in Korean-speaking children is largely complete by the latter half of age 3 (16, 17). Instead, it is best explained by the vowel composition of the two word lists: UTAP-2 samples only monophthongs, whereas Hi-DongDong samples both monophthongs and diphthongs, together with a wider range of onset–vowel combinations. Because monophthong production is mastered early, UTAP-2 quickly saturates and loses the ability to discriminate among older children; the more demanding vowel contexts in Hi-DongDong preserve measurable variance across a broader age span. A parallel, though far more subtle, pattern was seen for consonants, where Hi-DongDong PCC plateaued only about one year earlier than UTAP-2 (age 9 vs. 10; 99.1% vs. 99.2%). Taken together, these trajectories suggest that Hi-DongDong may provide broader coverage of the phonological system by capturing vowel production, including diphthongs, in addition to consonant accuracy. Rather than a source of disagreement, this broader construct coverage is a strength of the digital instrument, positioning it to assess consonant and vowel production jointly within a single administration. The clinical value of this expanded vowel coverage should nonetheless be confirmed in children with speech sound disorders, in whom vowel errors are more prevalent and more diagnostically relevant than in the typically developing sample studied here. Because the age-stratified analyses generated multiple estimates across individual ages and outcome measures, findings at specific ages should be interpreted as exploratory and hypothesis-generating rather than as independent confirmatory findings.

Two age-stratified recommendations follow from these findings. First, Hi-DongDong is most readily deployable in school-age children (7 years and older). In this range, bias was negligible (within ±1 percentage point) and LoA were narrow (approximately 8–24 percentage points for consonant metrics), indicating relatively close individual-level agreement between the two word lists even where ICC was attenuated by ceiling effects. This has direct practical significance: because UTAP-2 provides normative data through age 7, the present findings support further evaluation of Hi-DongDong as a potential complementary assessment option for older school-age children, for whom the reference instrument has no normative support. The low ICC values at ages 7 and 9 nonetheless warrant additional clinical judgment when these tools are used interchangeably at those specific ages. Second, in preschool-age children (2–6 years), the wide individual-level LoA (up to ∼30 percentage points for PCC, and larger still for the binary PWC metric) means that a single child's Hi-DongDong and UTAP-2 scores may differ substantially. In this range Hi-DongDong should not be treated as a stand-alone substitute for UTAP-2; concurrent administration, or use of Hi-DongDong as Supplementary Information alongside the reference instrument, is more appropriate. The particularly wide LoA for PWC across all ages reflects its word-level binary scoring, in which a single phoneme error is counted as a whole-word error, making it especially sensitive to differences in word-list composition between the two instruments.

### 4.1 Limitation

Several limitations should be considered. First, the age-specific sample sizes were unbalanced, and ICC estimates at ages 2 (*n* = 7) and 13 (*n* = 10) may be unstable. Second, this study enrolled only typically developing children; agreement between the two instruments in children with speech sound disorders must be established separately, particularly given the greater relevance of vowel and distortion errors in clinical populations. Additionally, eligibility as “typically developing” was based on caregiver report of the absence of language delay or health concerns rather than formal hearing screening or a standardized developmental checklist, and detailed recruitment procedures (e.g., sampling frame and recruitment sites) were not fully described; both should be reported more systematically in future studies to support replication and generalizability. Separately, one or more authors of the present study were also involved in the original Delphi-based development of the Hi-DongDong instrument (4); developer-led validation of one's own instrument is a recognized potential source of measurement or interpretation bias in instrument-validation research, independent of financial conflict, and this overlap should be considered when interpreting the present findings. In addition, the present study evaluated agreement between scores elicited by the Hi-DongDong and UTAP-2 word lists using manual clinician scoring; therefore, the findings should not be interpreted as evidence of the diagnostic accuracy or automated scoring performance of the Hi-DongDong digital system. Third, no prior work has established clinically acceptable thresholds for Bland–Altman LoA in this context, which constrains interpretation of whether the observed LoA ranges are clinically tolerable; defining a minimal clinically important range is an important priority for future research. In additional, residual diagnostics showed departures from normality in the tails and decreasing residual variance at higher fitted PCC values, consistent with the bounded 0%–100% scale and ceiling effects. Although sensitivity analyses yielded a similar overall pattern for the primary instrument-by-age interactions, these distributional characteristics should be considered when interpreting the model estimates. Finally, age was recorded in years rather than months, precluding finer-grained analysis of developmental change; future studies should track age in months to characterize articulatory and phonological development more precisely. Beyond addressing these limitations, the present age-specific agreement patterns provide concrete benchmarks for the next phase of Hi-DongDong development—the implementation of AI-powered automated scoring. The differential performance observed across preschool and school-age populations, and the identification of ages 4 and 7 as periods of genuine measurement divergence, will guide algorithm development priorities and define the performance thresholds against which automated scoring must be validated.

## 5 Conclusions

In 505 typically developing Korean children aged 2–13 years, Hi-DongDong and UTAP-2 were not statistically significantly different at the population level, yet their agreement at the individual level was strongly age-dependent. Interpreting ICC and Bland–Altman limits of agreement jointly proved essential: low ICC values at ages 9 and 12 were artifacts of ceiling-induced variance restriction rather than true disagreement, whereas the low ICC and wide limits of agreement at ages 4 and 7 reflected genuine measurement divergence during periods of rapid or transitional phonological development. Hi-DongDong also sustained discriminative sensitivity for both consonants and vowels—including diphthongs—across a wider developmental range than the monophthong-only UTAP-2, indicating broader phonological coverage within a single administration. Clinically, Hi-DongDong is a viable standardized alternative for articulation assessment in school-age children (7 years and older), for whom UTAP-2 lacks normative data, but in preschool-age children it should be used alongside, rather than in place of, the reference instrument. These age-stratified findings provide the benchmarks needed to guide and validate the forthcoming AI-powered automated scoring of Hi-DongDong.

## Data Availability

The raw data supporting the conclusions of this article will be made available by the authors, without undue reservation.
